# Effect of Sugarcane Cultivars Infected with Sugarcane Yellow Leaf Virus (ScYLV) on Feeding Behavior and Biological Performance of *Melanaphis sacchari* (Hemiptera: Aphididae)

**DOI:** 10.3390/plants10102122

**Published:** 2021-10-06

**Authors:** Luiz Eduardo Tilhaqui Bertasello, Michele Carmo-Sousa, Nathalie K. Prado Maluta, Luciana Rossini Pinto, João R. Spotti Lopes, Marcos Cesar Gonçalves

**Affiliations:** 1 School of Agricultural and Veterinarian Sciences—FCAV, São Paulo State University—UNESP, Jaboticabal 17884-900, Brazil; luiz.bertasello@unesp.br (L.E.T.B.); luciana.rossini@sp.gov.br (L.R.P.); 2 Department of Entomology and Acarology, Escola Superior de Agricultura Luiz de Queiroz (ESALQ), University of São Paulo, Piracicaba 13418-900, Brazil; m.sousatimossi@gmail.com (M.C.-S.); nathaliepradomaluta@gmail.com (N.K.P.M.); 3 Sugarcane Research Centre, Instituto Agronômico de Campinas—IAC, Ribeirão Preto 14001-970, Brazil; 4 Crop Protection Research Centre, Instituto Biológico—IB, São Paulo 04014-002, Brazil

**Keywords:** aphid performance, electrical penetration graph, probing behavior, plant virus resistance, *Saccharum* spp.

## Abstract

*Sugarcane yellow leaf virus* (ScYLV), *Polerovirus*, *Luteoviridae*, is one of the main viruses that infect sugarcane worldwide. The virus is transmitted by the aphid *Melanaphis sacchari* in a persistent, circulative manner. To better understand the interactions between ScYLV, sugarcane genotypes and *M. sacchari*, we explored the effect of sugarcane cultivars on the feeding behavior and biological performance of the vector. The number of nymphs, adults, winged, total number of aphids and dead aphids was assayed, and an electrical penetration graph (EPG) was used to monitor the stylet activities. Multivariate analysis showed changes in the vector’s behavior and biology on cultivars, identifying specific groups of resistance. In the cultivar 7569, only 5.5% of the insects were able to stay longer on sustained phloem ingestion, while in the other seven cultivars these values varied from 20% to 60%. *M. sacchari* showed low phloem activities in cultivars 7569 and Bio266. Overall, cultivar 7569 showed the worst biological performance of aphids, with the insects presenting mechanical difficulties for feeding and a shorter duration of the phloem period, and thus being considered the most resistant. We conclude that ScYLV virus infection in different sugarcane cultivars induced specific changes in the host plant, modifying the behavior of its main vector, which may favor or impair virus transmission.

## 1. Introduction

*Sugarcane yellow leaf virus* (ScYLV), genus *Polerovirus*, family *Luteoviridae*, responsible for the yellow leaf disease (YLD) of sugarcane, is restricted to the plant phloem [1] and efficiently transmitted by the aphid *Melanaphis sacchari* (Hemiptera: Aphididae) in a persistent, circulative and non-propagative manner [2]. Leaf yellowing is the characteristic symptom of ScYLV infection, starting from the midrib of the abaxial leaf surface and, in advanced stages, spreading through the leaf blade, while the midrib of the adaxial surface presents a reddish color [3], shortening of the stalks’ internodes [4] and accumulation of sucrose in the phloem [5]. The virus has become endemic in the main producing countries [6] and remains a major concern for sugarcane producers [7], causing field losses of 20 to 60% in susceptible cultivars [8,9,10], and of up to 14% in sugar net yield [11].

Viruses can modify directly or indirectly the behavior of their vectors to increase the chances of transmission to new hosts [12]. These behavioral changes can occur in different ways, depending on whether they result from the presence of the virus in the vector body (direct effect), or mediated by physiological changes in the host plant (e.g., sugar content and leaf color) as a result of infection by the pathogen, which can modulate the behavior and performance of the vector in the host (indirect effect) [13]. These changes allow the virus to influence the vector’s landing, performance, feeding behavior and stylet activities in the host plant [14]. Thus, the transmission of plant viruses by their arthropod vectors is mediated by specific interactions between the plant, the virus and the vector [15].

One of the determining factors in viral epidemiology is the interactions between viruses and their vectors [16], and the advance in knowledge between these interrelationships is essential in the development of new disease management strategies and in the selection of resistant cultivars. In this context, hemipterans are responsible for more than 70% of all plant viruses transmitted by insects, featuring the high number of vector species and their economic importance [17,18].

Several physicochemical changes can occur in host plants due to viral infection, causing changes in the volatile composition, color, hormonal, nutritional and water status of the plants [19,20], which can modulate the behavior of host choice by insect vectors and may favor the spread of the virus to new plant hosts [14,21,22,23]. Persistent, circulative-transmitted viruses can induce changes in infected plants [21], which can alter the feeding behavior of these insects [21,24] and may directly interfere with the acquisition and inoculation processes of the pathogen [14,25]. Additionally, any physiological or morphological modification in plants can interfere in the feeding behavior of insects and, consequently, in their biological performance and virus transmission.

The feeding behavior activities of sap-sucking insects can be monitored in real time by the electrical penetration graph (EPG) technique, an important tool enabling the study of an aphid’s stylet activities [26]. Hence, EPG is considered a fundamental tool for development of the research encompassing insect vectors and the transmission of plant pathogens [17], with excellent efficiency in plant–virus–vector interaction studies, incorporating the characterization of host plant resistance to the insect vector [27].

The different EPG waveforms have been characterized after numerous observations on the feeding behavior of aphids [26,28,29,30], and the different stylet activities of these insects in host plants are associated with biological activities. EPG waveforms have been characterized based on the amplitude, frequency and origin of the electrical signal (resistance or electromotive force) [31,32].

The research hypothesis of this work is that ScYLV-infected sugarcane cultivars possibly possess specific features modified by the virus and intrinsic degrees of resistance, which can affect the biology and feeding behavior of *M. sacchari* in a distinct way, allowing the identification of sources of resistance to the vector. Hence, the objective of this work was to determine the effect of eight ScYLV-infected sugarcane cultivars on the feeding behavior and biological performance of *M. sacchari*, and to identify cultivars that present some type of resistance to the vector and consequently are expected to reduce the virus transmission rate in the field.

## 2. Results

### 2.1. Effects of ScYLV-Infected Sugarcane Cultivars on the Life History of M. sacchari

**Figure 1:**
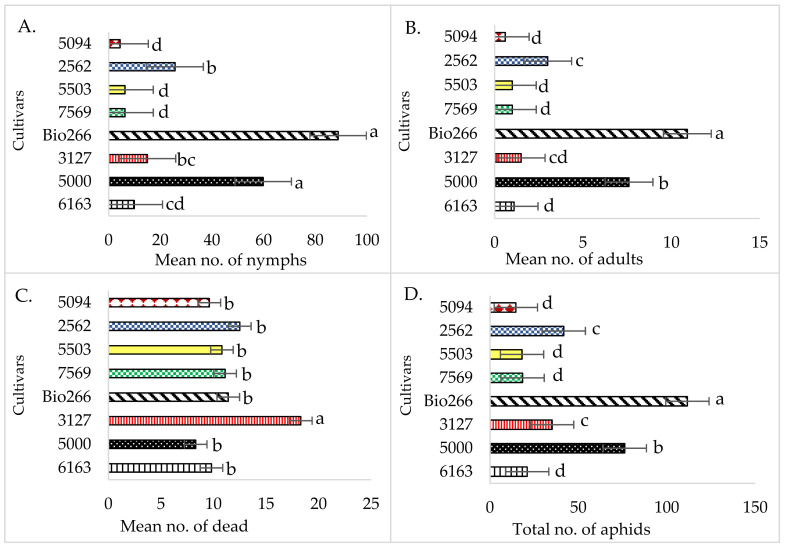
Effect of sugarcane cultivars infected with ScYLV on the biological performance of *M. sacchari*. Mean ± SE that share the same letter for each parameter are not significantly different (*p* > 0.05). Statistical com-parisons between treatments for each parameter were done using the non-parametric Kruskal–Wallis test or parametric ANOVA/Fisher test (**A**–**D**).

Differences were identified in all biological parameters evaluated (*p* < 0.05) between cultivars (Figure 1). From a Fisher’s test comparison, it was observed that the number of nymphs was significantly higher in cultivars Bio266 and 5000 than in the other treatments; lower in cultivars 5094, 5503 and 7569; and intermediate in cultivars 2562, 3127 and 6163 (*p* = 0.04) (Figure 1A).

The number of adults was significantly higher in cultivar Bio266, followed by cultivar 5000; lower in cultivars 5094, 5503, 7569 and 6163; and intermediate in cultivars 2562 and 3127 (Figure 1B). A similar trend was observed for the total number of aphids (Figure 1D). The highest average mortality rate of *M. sacchari* was observed in cultivar 3127, showing significant differences in relation to the other cultivars (*p* = 0.01), (Figure 1C).

Kruskal–Wallis non-parametric analysis (*p* > 0.05) were applied to the mean index of winged insects data, which showed differences with superior performance in cultivars 5000 and 2562, with means of 0.52 and 0.50, respectively, in comparison with the cultivars 5503, 5094 and 7569 (data not shown). The other cultivars did not differ from each other.

**Figure 2:**
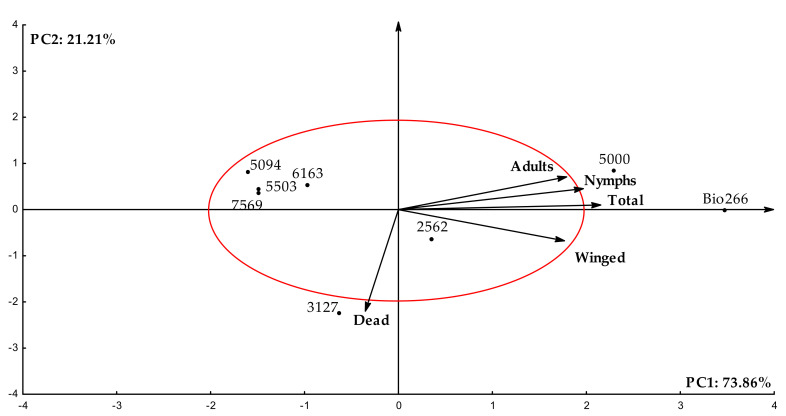
Biplot with dispersion of eight sugarcane cultivars regarding biological performance in the reproduction of wingless in-sects under no-choice conditions, analyzed seven days after release. Data submitted to exploratory multivariate principal component analysis (PCA).

Through the multivariate exploratory analysis by principal components (PCA) regarding the dispersion pattern of sugarcane cultivars, the original variance contained in the data was 95.07% in the first two PCAs, fitting in the criterion proposed by Kaiser [33] (Table 1 and Figure 2). With the application of the two components, it was possible to correlate their factors and the variables, as the values were higher than 0.7. On PCA1, the variables that stood out with the greatest potential for discrimination explained 73.86% of the contained variance, whereas on PCA2 the representation was 21.21% of the original contained variance (Table 1).

By the dispersion presented in the two components formed for the eight sugarcane cultivars (PCA1 and PCA2), showed in the biplot graph, three cultivars are located outside the ellipse formed from −2 to 2, indicating cultivars with specific biological performance features: Bio266, 5000 and 3127. The other five cultivars, located inside the ellipse, are considered without any specific performance feature; i.e., without major differences regarding the biological performance of wingless *M. sacchari* (Figure 2). Cultivars Bio266 and 5000 were discriminated with the best aphid performance, presenting the highest averages for number of nymphs, adults, winged and total aphids. However, considering the number of deaths, cultivar 5000 presented the lowest mortality rate, suggesting a greater acceptance of the cultivar by the insects (Figure 1C). On the other hand, cultivar 3127 showed the highest aphid mortality rate (18.3) by the end of the seventh day of recordings. Thus, it was possible to identify specific groups of interest regarding the biological performance of the vector, based on the lower or higher reproductive capacity of *M. sacchari* in cultivars infected with ScYLV.

**Figure 3:**
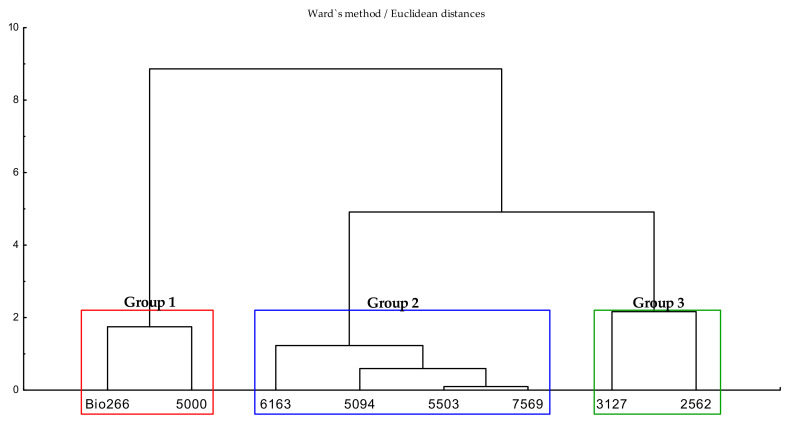
Dendrogram based on the Euclidean distance between eight sugarcane cultivars regarding the biological performance of wingless M. sacchari under no-choice conditions, analyzed seven days after release. Data submitted to multivariate ex-ploratory cluster analysis using Ward’s method.

Based on the Euclidean distance between the different parameters assayed, the cutoff point 2.18 was adopted and Ward’s hierarchical clustering method was applied, with the results illustrated in the dendrogram showed in Figure 3. The cultivars were divided into three specific groups, namely, Group 1, Group 2 and Group 3. The formation of the three distinct groups, based on the similarity between the resulting parameters of the cultivars, corroborates the results found in the biplot distribution by the principal components method (Figure 2).

Group 1, formed by cultivars Bio266 and 5000, showed the highest biological performance index. This result corroborates the other analyzes, where the highest means were observed for the variables number of nymphs, adults, winged and total insects. The similarity formed within Group 2, encompassing cultivars 6163, 5094, 5503 and 7569 (Figure 3), all of them within the ellipse ranging from −2 to 2, with the lowest reproduction rates, number of nymphs, adults, winged and total insects, which confirms the results in the biplot (Figure 2). Group 3 (3127 and 2562) was strongly characterized by the highest mortality rate of *M. sacchari*, 18.3 and 12.5, respectively.

### 2.2. Stylet Activities and Probing Behavior of M. sacchari in ScYLV-Infected Sugarcane Cultivars

In the EPG experiment, differences were observed among cultivars in the feeding behavior and stylet activities of *M. sacchari* during the 8 hours of recording. Regarding the proportion of individuals that produced a specific type of waveform (PPW), no differences were observed on phloem waveforms E1 (x^2^ = 12.25; df = 7; *p* = 0.09) and E2 (x^2^ = 12.37; df = 7; *p* = 0.09), and the percentages of individuals that were able to perform activities in these vessels ranged from 16 to 66%.

**Figure 4:**
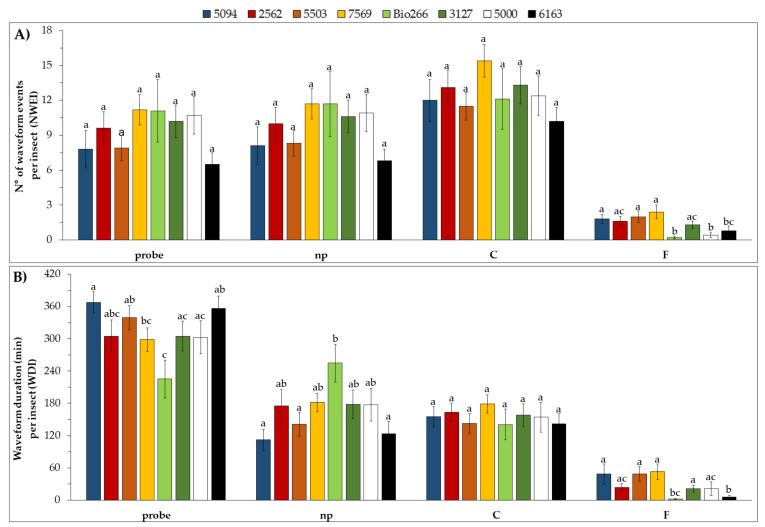
Mean ± SE of the number of waveform event per insect (NWEI) (**A**) and the total waveform duration per insect (WDI) (**B**) of the non-phloematic parameters of non-viruliferous M. sacchari on ScYLV-infected sugarcane cultivars during 8 h of recording. The columns and bars represent the means and the standard error of the mean for each variable, respectively. Statistical comparisons between treatments (in the same EPG parameter) were conducted by a Tukey test (for Gaussian variables) or Kruskal–Wallis test (for non-Gaussian variables). Means that share the same letter for each variable are not significantly different (*p* > 0.05). Waveform C: stylet pathway; np: non-probing and probe; F: mechanical disturbance during feeding.

Significant differences were observed in the proportion of individuals that performed the waveform (PPW) F (x^2^ = 31.01; df = 7; *p* < 0.01), which indicates mechanical difficulty for penetration of the stylets during the probing behavior. This was observed in cultivars 5000 (22.2%), Bio266 (23.5%) and 6163 (27.8%), in which a smaller proportion of individuals performed a waveform F when, compared to cultivars 5094 (80%), 2563 (64.7%), 5503 (60%), 7569 (73.7%) and 3127 (76.5%). Moreover, differences were observed on the PPW corresponding to sustained phloem ingestion (E2 > 10 min) (x^2^ = 16.35; df = 7; *p* = 0.01). E2s was markedly lower in cultivar 7569, in which only 5.5% of the aphids were able to ingest phloem sap for more than 10 min, whereas in the other treatments the percentage ranged from 20 to 61% (Supplementary Material Table S3). Regarding the non-sequential variables, differences were detected in the number (NWEI) of G, F, pd and E2s, and in the total duration of probing, non-probing, G, F, pd and E2. (Figure 4).

On the other hand, in the xylem phase (waveform G), the aphids performed activities in the xylem (NWEI, waveform G) less often in cultivar Bio266 (H = 17.09; df = 7; *p* = 0.02), and the total duration (WDI) of this phase was also shorter in this cultivar (H = 24.10; df = 7; *p* < 0.01). The number of brief intracellular punctures (waveform pd) was higher in cultivar 7569 compared to cultivars 5503, Bio266, 3127, 5000 and 6163, and lower in cultivar Bio266 compared to cultivars 5094, 2562, 5503 and 7569 (H = 24.37; df = 7; *p* < 0.01). The total duration (WDI, waveform pd) basically followed the same trend, being higher in cultivar 7569 compared to 5503, Bio266, 3127 and 5000, and shorter in cultivar Bio266 than in cultivars 5094, 2562, 7569 and 6163 (H = 21.29; df = 7; *p* < 0.01) (Supplementary Material Table S1).

The number (NWEI) of F was significantly higher in cultivars 7569, 5503, 5094, 2562 and 3127, and lower in cultivars Bio266, 5000 and 6163 (H = 29.34; df = 7; *p* < 0.01). In cultivars Bio266 and 6163, the insects spent less time performing activities associated with waveform F; that is, they had less mechanical difficulty with the stylets than in the other treatments (H = 29.56; df = 7; *p* < 0.01) (Figure 4).

The total duration of the probes (WDI) was shorter in cultivar Bio266 (H = 14.88; df = 7; *p* = 0.04) compared to cultivars 5094, 5503 and 6163, and the total duration of non-probing was lower in cultivars 5094, 6163 and 5503 compared to cultivar Bio266 (H = 15.15; df = 7; *p* = 0.03) (Figure 4).

**Figure 5:**
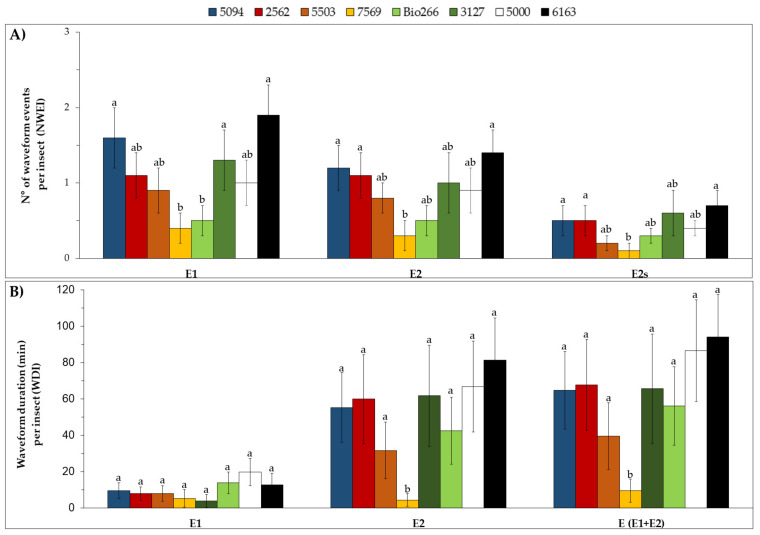
Mean ± SE of the number of waveform event per insect (NWEI) (**A**) and the total waveform duration per insect (WDI) (**B**) of the phloematic parameters of non-viruliferous *M. sacchari* on ScYLV-infected sugarcane cultivars during 8 h of re-cording. The columns and bars represent the means and the standard error of the mean for each variable, respectively. Statistical comparisons between treatments (in the same EPG parameter) were conducted with a non-parametric Kruskal–Wallis test. Means that share the same letter for each variable are not significantly different (*p* > 0.05). Waveform E1: phloem salivation; E2: phloem ingestion; and E2s: sustained phloem ingestion >10 min.

Regarding the parameters associated to the phloem, differences were observed in the number (NWEI) of E1 (H = 14.79; df = 7; *p* = 0.04), E2 (H = 13.83; df = 7; *p* = 0.04) and E2s (H = 14.35; df = 7; *p* = 0.04), and insects performed salivation and ingestion activity less often in cultivars 7569 and Bio266. The duration of the phloem sap ingestion period (WDI waveform E2) in cultivar 7569 was significantly shorter than in the other cultivars (H = 14.70; df = 7; *p* = 0.04). In addition, the total duration of E (E1 + E2) was also significantly shorter in this cultivar (H = 19.92; df = 7; *p* = 0.04), (Figure 5).

No significant differences were observed for any sequential variable (Supplementary Material Table S2).

## 3. Discussion

The development of sugarcane cultivars resistant to ScYLV and to its main vector, the aphid *M. sacchari*, are essential for sugarcane breeding programs, representing an important step to ensure productivity for the sugar and alcohol production chain. One of the determining factors in viral epidemiology is the interaction between the pathogen and its vector [34]; consequently, the advance in the knowledge of the relationships between them is essential for the development of effective management strategies.

This research evaluated the effect of genotypic variation of sugarcane cultivars infected with ScYLV on the behavior and biology of *M. sacchari,* aiming to verify whether the viral infection, combined with the phenotypic features of each cultivar, influence the insect reproduction and feeding behavior, and consequently on the spread of the virus to other plants.

In general, plants infected with persistent, circulative-transmitted viruses tend to be more attractive and nutritionally richer than non-infected plants, in order to attract the vector and induce it to feed for a longer time, and consequently acquire the virus more efficiently [14,35]. Virus infection may also produce changes in canopy color and plant volatile compounds emission that favors vector attraction and landing on the crop [36,37]. For instance, the analysis of the volatile compounds of Citrus tristeza virus-infected tolerant citrus varieties indicated a higher presence of monoterpenes, known to be the main components showing deterrent properties toward viruses and insect vectors [38]. Mauck et al. [37] showed that plants induce elevated emissions of a plant volatile blend that increases their attractiveness to alate aphids. These aspects are correlated with the co-evolution between the vector and the virus [39,40] and infers an important role in the transmission mode, influencing virus-mediated changes in the vector’s behavior [39] and biological performance, optimizing virus dissemination [40,41].

Our study, through controlled biological assays and feeding behavior evaluated by EPG, allowed the identification and characterization of specific heterogeneous groups of sugarcane cultivars regarding resistance to *M. sacchari*. Cultivars Bio266 and 5000 showed the best biological performance of *M. sacchari*, showing the highest rates of vector reproduction, and consequently, a significant colony increase in their leaves.

On the other hand, cultivars 5094, 5503 and 7569, and to an intermediate degree, cultivars 3127 and 2562, showed signs of resistance in the *M. sacchari* reproductive parameters. In addition, higher mortality was observed in cultivar 3127, which can reduce the number of vectors and, consequently, the transmission rate of ScYLV over the generations (Figure 1). These results show that these varieties have specific features capable of modifying the colonization behavior of the vectors; thus, it can be recommended for planting where secondary transmission of ScYLV by aphids in the field is a problem, or even be used as sources of resistance genes in sugarcane breeding programs.

The reproductive potential rate is an indicator used to assess aphid resistance mechanisms, and low values can be an indicative of plant resistance [20,23,42]. In recent studies of the interaction between *M. sacchari* and sorghum genotypes, this rate has been reported with variable effect, confirming the resistance mechanism by antibiosis [43,44]. According to Fartek et al. [45], the knowledge in the plant–virus–vector interactions combined with genomic mapping and other techniques based on feeding behavior, aphid biology and phenotypic expression of this interaction, are effective ways to evaluate and select genotypes with durable resistance to ScYLV. In addition, Smith [42] states that the search for and use of cultivars resistant to virus vector insects is an important tool for environmental management and is economically advantageous when combined with other control techniques.

In our work, the factor–variable correlations obtained through multivariate analysis, as well as the plotting of the respective indexes and the identification of specific groups of interest, according to insect colonization and their respective survival and reproduction rates (Figure 2 and Figure 3), confirmed varying degrees of resistance to the vector of each sugarcane cultivar.

The application of multivariate analyses has also been widely used in other studies with *M. sacchari*, such as the one reported by Nibouche et al. [46], who studied the genetic variability of *M. sacchari* in different locations, with more than 540 plant species, including sugarcane, sorghum, and corn, identifying the similarity and the formation of genetically superior populations. Using the same multivariate technique, Park et al. [47] analyzed the herbivory and discriminated sorghum genotypes not infested from those colonized by the aphid *M. sacchari*. Additionally, Rodríguez-Vélez et al. [48] also succeeded in studying the behavior of *M. sacchari* under different environmental conditions, through principal component analysis and factor-variable correlation.

Due to viral infections, physicochemical changes occur in plants, influencing the spread of the virus between different host species, making the plant more attractive [14,22,23] or repelling insect vectors, interfering with its biological performance [21,24,49]. Combined with biological performance assays, the EPG technique allows the identification of possible changes in the feeding behavior and stylet activity of insects, giving indications of putative characteristics inherent to the cultivars associated with ScYLV infection, which may indicate resistance to *M. sacchari*. The activities associated with phloem vessels are remarkably important in this study, because ScYLV is a virus limited to phloem vessels and transmitted in a persistent, circulative manner, thus requiring long periods of vector feeding (salivation and ingestion) for its effective transmission [17]. In this case, the aphids must reach the phloem to release the viral particles and inoculate a healthy plant, along with the watery saliva excreted during the salivation phase (E1 waveform) [50], and, on the other hand, remain for long periods ingesting phloem sap (E2 waveform) for the acquisition of ScYLV.

The results obtained from the aphid’s feeding behavior assays revealed that cultivar 7569 most effectively affected the behavior of *M. sacchari*, since the insects performed a greater number and duration of waveforms F, which is related to the mechanical difficulty during the probing behavior, indicating possible resistance factors in the cultivar. Furthermore, in this cultivar, the aphids showed a greater number and duration of pds, fewer E1, E2 and E2s, and a shorter duration of the phloem phase (E2 and E(E1 + E2)). This behavior indicates that the insects performed a greater number of brief intracellular punctures (pds), but due to the mechanical difficulty (waveform F), possibly linked to the morphological characteristics of this cultivar, they did not succeed in feeding from the phloem vessels, which, associated with the biological data, indicates a higher degree of resistance of this cultivar. In this case, similarly to what was observed by Ma et al. [51] and Tetreault et al. [52], the host negatively influences the feeding behavior of the vector and consequently reduces its fecundity.

In cultivars 5503, 5094, 2562 and 3127, the insects found mechanical difficulty in feeding, as evidenced by the greater number and duration of the waveform F; however, they were able to reach and normally feed on the phloem. On the other hand, cultivar Bio266 showed the best biological performance, evidenced by the higher number of aphids in the plants (nymphs and adults), as well as by the smaller mechanical difficulty of the stylets (waveform F). However, it was also the cultivar in which aphids performed a small number of activities associated with the phloem (E1 and E2), but without affecting the duration of these phases, in addition to spending more time in np and to a shorter total duration of probes. However, the contrasting data between biology and feeding behavior do not allow to conclude whether cultivar Bio266 is in fact susceptible or not.

Taking into account the results of the biological performance and feeding behavior together, cultivar 7569 showed the highest resistance to *M. sacchari*, as the biological performance (number of nymphs, adults, and total insects) was worse and the insects presented mechanical difficulty to feed and a shorter duration of the phloem period, mainly in the ingestion phase. Cultivar 6163, known to be susceptible to ScYLV infection, despite not showing good biological performance of aphids, proved to be attractive in terms of feeding, as evidenced by less mechanical difficulty to reach the phloem and a longer feeding period in the phloem vessels.

The poorer feeding performance, along with the decrease in the number of *M. sacchari* offspring, provides evidence of sugarcane cultivars resistant to the vector, which can affect the transmission of ScYLV. However, other studies involving free-choice trials using healthy and infected plants of each cultivar are needed to estimate more attractiveness and repellency factors in the existing interactions among sugarcane cultivars, ScYLV and *M. sacchari*. Overall, our results effectively contribute on the identification and characterization of sugarcane genotypes that offer sources of resistance to the main ScYLV vector, the aphid *M. sacchari*.

## 4. Materials and Methods

### 4.1. Aphid Colony and Test Plants

Pre-sprouted seedlings (PSS) of eight sugarcane cultivars were used: IACSP95-5094, IACCTC05-2562, IACSP01-5503, IACSP96-7569, IACBio-266, IACSP01-3127, IACSP95-5000 and SP71-6163; the last one is markedly susceptible to ScYLV, and therefore used as the reference. The first seven cultivars were released by the Agronomic Institute of Campinas (IAC) Sugarcane Breeding Program and are well accepted by the cane growers in Brazil. IACSP95-5094, IACCTC05-2562, IACSP01-5503, IACSP96-7569, IACSP01-3127 and IACSP95-5000 are known for their high sucrose yield and adaptation to different Brazilian soil and climate conditions, whilst IACBio-266 is an energycane cultivar, recently in demand from the sugarcane market for ethanol production. As there was a lack of information regarding *M. sacchari* biology and feeding behavior on these cultivars, they were selected for this work. After germination and development of PSS, all plants were grown in a greenhouse for three months, where viruliferous populations of *M. sacchari* were released every 15 days, allowing the viral infection to occur uniformly.

The viruliferous colony of *M. sacchari* was reared on detached leaves of sugarcane cultivar IACSP95-5000 infected with ScYLV and kept in test tubes with 1% agar solution, in a growth chamber (12 L:12 D, 29 ± 1 °C). The sugarcane leaves were changed every 6 ± 1 day. A non-viruliferous population of *M. sacchari* was reared separately on healthy plants (non-infected sugarcane plants) and the absence of the virus was confirmed by RT-PCR analysis [53].

To facilitate the presentation and interpretation of the data, the name of the cultivars used in this research will be referred to only by their respective numbers.

### 4.2. Effects of ScYLV-Infected Sugarcane Cultivars on the Life History of M. sacchari

The biological performance assays were carried out at the Agronomic Institute (IAC) Sugarcane Research Centre in Ribeirão Preto, SP, Brazil, following the experimental model described by Fartek et al. [45]. Ten replicates were individually done for each of the eight cultivars, with leaves kept in properly identified test tubes, containing 1% agar solution, and covered with sheer fabric (voile), preventing the insect’s escape and allowing internal ventilation. In each replicate, five apterous adults of non-viruliferous *M. sacchari* were released and plants were kept in a growth chamber (12 L:12 D, 29 ± 1 °C). Seven days after the aphids’ release, the total number of aphids, the number of nymphs and adults, the number of alates as well as the total number of dead aphids (adults and nymphs) were counted.

### 4.3. Stylet Activities and Probing Behavior of M. sacchari in ScYLV-Infected Sugarcane Cultivars

The stylet activities and feeding behavior of *M. sacchari* was evaluated using the EPG technique, as described by Tjallingii [29] and Carmo-Sousa et al. [54], with adaptations for the sugarcane plant architecture.

To prepare the insects for the EPG assay, apterous adults of *M. sacchari* were immobilized individually using a vacuum chamber under a dissecting microscope. Then, a gold wire (3 cm length, 18 μm in diameter; EPG Systems, Wageningen, The Netherlands) was attached to the aphid’s pronotum with a small droplet of conductive silver paint glue (Pelco Colloidal Silver Liquid; Ted Pella Inc., Redding, CA, USA). The opposite end of the gold wire was glued to a thin copper wire (2 cm length), which was connected to the EPG probe. The output electrode was a copper post (10 cm long, 2 mm diameter) inserted into the soil of the plant container to close the circuit. After a 1 h starvation period, each aphid was placed individually on the abaxial surface of a sugarcane leaf and then connected to a DC-EPG device.

The EPG waveforms were recorded for 8 h inside a Faraday cage (for isolation from electrical noise) in a climate-controlled room (25 ± 1 °C) using a Direct Current (DC) eight-channel EPG device, model Giga−8 d, with Stylet+ for Windows software (EPG Systems, The Netherlands) [29]. A total of 17–20 replicates were performed on eight sugarcane cultivars.

The EPG data were analyzed according to the waveforms described for aphids by Tjallingii [29] and van Helden and Tjallingii [30]: non-probing (np); intercellular stylet pathway activities (C); intracellular punctures during stylet pathway phase (pd); phloematic phase-salivation into phloem sieve elements (E1); passive phloem-sap ingestion (E2); active intake of xylem sap (G); and derailed stylet mechanics (F).

The output of 8 h EPG recordings given by the EPG-Excel Data Workbook 5.0 of Sarria et al. [55] for each aphid were used to calculate the treatment mean for each EPG’s sequential and non-sequential variables. The selected EPG variables (mean ± SE) were calculated and compared between treatments as previously described by Backus et al. [56]: PPW—proportion of individuals that generated a particular waveform type; NWEI—number of waveform events per insect; WDI—total waveform duration (min) per insect; and sequential variables—Time to 1st probe from start of EPG; Time from start of EPG to 1st E; Time from 1st probe to 1st E; Time to from start of EPG 1st sustained E2 (10 min).

### 4.4. Statistical Analysis

Prior to statistical analysis, normality, according to the Shapiro–Wilk test [57], and homogeneity of variance were checked out. The EPG data were compared by a parametric Tukey’s test (*p* < 0.05) (for Gaussian distribution), and data which did not show a normal distribution were transformed by √ (x + 1) or ln (x + 1). For the data that even after transformation did not follow a normal distribution, the nonparametric Kruskal–Wallis H-test (*p* < 0.05) (for non-Gaussian distribution) was performed, followed by pairwise comparison. A chi-square test was used to analyze the proportion of individuals that produced a specific waveform type (PPW). Statistical analysis was conducted using the IBM SPSS Statistics software package, version 22.0 [58].

Biological parameters of *M. Sacchari* were analyzed by ANOVA and Fischer´s test (*p* < 0.05) (for Gaussian distribution), and a nonparametric Kruskal–Wallis test (*p* < 0.05) (for non-Gaussian distribution) for data that after transformation did not follow a normal distribution. The same data were collected in an exploratory multivariate analysis by clusters and principal components (PC). The results of the cluster analysis were plotted on dendrogram-type graphs, exploring existing hierarchical levels of similarity, and defining specific groups according to Ward’s criterion. A PC analysis was based on the selection of components that separate eigenvalues above 1.0, according to the Kaiser criterion [59], with the results plotted on biplot graphs.

Statistical analysis was conducted using the software SAS version 9.3 (Sas Institute) [60] and Statistica version 7.0 (Statsoft) [61].

## Figures and Tables

**Table 1:** Factor-variable correlations in the first two main components for eight sugarcane cultivars regarding the biological performance of *M. sacchari* under no-choice conditions. Data submitted to multivariate exploratory analysis by principal components (PCA).

Variables	PC1	PC2
Mean no. of nymphs	0.98	0.04
Mean no. of adults	0.98	0.08
No. winged	0.86	−0.29
No. dead	−0.12	−0.98
Total	0.99	0.01
Eigenvalues	3.69	1.06
Variance (%)	73.85	21.21
Accumulated variance (%)	73.85	95.06

## Supplementary Materials

The following are available online at https://www.mdpi.com/article/10.3390/plants10102122/s1, Table S1: Mean (±SEM) of non-sequential EPG variables for 8-h recordings of the probing behavior of *Melanaphis sacchari* on sugarcane cultivars infected with sugarcane yellow leaf virus (ScYLV), Table S2: Mean (±SEM) of sequential EPG variables for 8-h recordings of the probing behavior of *Melanaphis sacchari* on sugarcane cultivars infected with sugarcane yellow leaf virus (ScYLV), Table S3: Proportion of *Melanaphis sacchari* that produced a specific waveform type (PPW) on sugarcane cultivars infected with sugarcane yellow leaf virus (ScYLV) during 8-h recording.
