# Supplementary material for: Effect of Sugarcane Cultivars Infected with Sugarcane Yellow Leaf Virus (ScYLV) on Feeding Behavior and Biological Performance of Melanaphis sacchari (Hemiptera: Aphididae)
Source: Plants (Basel). 2021 Oct 6;10(10):2122. doi: 10.3390/plants10102122 (PMC8537889; doi:10.3390/plants10102122)
Supplement: Supplementary file 1 [file plants-10-02122-s001.zip › plants-1393913-supplementary.pdf]

Effect of sugarcane cultivars infected with Sugarcane yellow leaf virus (SCYLV) on feeding behavior and biological performance of *Melanaphis sacchari* (Hemiptera: Aphididae)

Luiz Eduardo Tilhaqui Bertasello [1], Michele Carmo-Sousa [2], Nathalie K. Prado Maluta [3], Luciana Rossini Pinto [1,4], João R. Spotti Lopes [3], Marcos Cesar Gonçalves [1,5*]

1 São Paulo State University - UNESP. School of Agricultural and Veterinarian Sciences - FCAV, Jaboticabal, São Paulo, 17.884-900 Brazil; luiz.bertasello@unesp.br

2 Fund for Citrus Protection - FUNDECITRUS - Araraquara, São Paulo, 14.807-000, Brazil; m.sousatimossi@gmail.com

3 University of São Paulo - ESALQ. Dep. of Entomology and Acarology, Piracicaba, São Paulo, 13.418-900, Brazil; nathaliepradomaluta@gmail.com (N.K.P.M.); jrslopes@usp.br (J.R.S.L.)

4 Sugarcane Research Centre, Instituto Agronômico de Campinas - IAC, Ribeirão Preto, São Paulo, 14.001-970, Brazil; luciana.rossini@sp.gov.br

5 Crop Protection Research Centre - Instituto Biológico - IB, São Paulo, 04.014-002, Brazil; marcos.goncalves@sp.gov.br

* Correspondence: jrslopes@usp.br (J.R.S.L.); marcos.goncalves@sp.gov.br (M.C.G.); Tel.: +55-11-50871767

Table S1. Mean (± SEM) of non-sequential EPG variables for 8-h recordings of the probing behavior of *Melanaphis sacchari* on sugarcane cultivars infected with sugarcane yellow leaf virus (ScYLV).

EPG Variable	5094 n = 20	2562 n = 17	5503 n = 20	7569 n = 18	Bio266 n = 17	3127 n = 17	5000 n=18	6163 n=18	P-value[b]	H or Z* value
**NWEI[a]**										
**Probe**	7.8 ± 1.6 a	9.6 ± 1.4 a	7.9 ± 1.1 a	11.2 ± 1.3 a	11.1 ± 2.7 a	10.2 ± 1.4 a	10.7 ± 1.6 a	6.5 ± 1.0 a	0.13	11.28
**np**	8.1 ± 1.6 a	10.0 ± 1.4 a	8.3 ± 1.1 a	11.7 ± 1.3 a	11.7 ± 2.8 a	10.6 ± 1.4 a	10.9 ± 1.6 a	6.8 ± 1.0 a	0.08	1.85*
**C**	12.0 ± 1.8 a	13.1 ± 1.5 a	11.5 ± 1.2 a	15.4 ± 1.4 a	12.1 ± 2.6 a	13.3 ± 1.6 a	12.4 ± 1.7 a	10.2 ± 1.2 a	0.40	1.06*
**G**	2.2 ± 0.5 ab	1.6 ± 0.4 ab	1.6 ± 0.3 ab	2.1 ± 0.4 b	0.6 ± 0.2 c	1.2 ± 0.4 ac	1.1 ± 0.3 ac	2.0 ± 0.3 b	0.02	17.09
**F**	1.8 ± 0.4 a	1.6 ± 0.4 ac	2.0 ± 0.5 a	2.4 ± 0.6 a	0.2 ± 0.1 b	1.3 ± 0.3 ac	0.4 ± 0.2 b	0.8 ± 0.4 bc	< 0.01	29.34
**pd**	48.3 ± 7.6 ab	52.6 ± 11.2 ab	41.4 ± 8.5 a	69.7 ± 8.2 b	24.0 ± 9.3 c	37.4 ± 7.1 ac	32.8 ± 10.4 ac	37.6 ± 6.6 ac	< 0.01	24.37
**E1**	1.6 ± 0.4 a	1.1 ± 0.3 ab	0.9 ± 0.3 ab	0.4 ± 0.2 b	0.5 ± 0.2 b	1.3 ± 0.4 a	1.0 ± 0.3 ab	1.9 ± 0.4 a	0.04	14.79
**E2**	1.2 ± 0.3 a	1.1 ± 0.3 a	0.8 ± 0.2 ab	0.3 ± 0.2 b	0.5 ± 0.2 ab	1.0 ± 0.4 ab	0.9 ± 0.3 ab	1.4 ± 0.3 a	0.04	13.83
**E2s**	0.5 ± 0.2 a	0.5 ± 0.2 a	0.2 ± 0.1 ab	0.1 ± 0.1 b	0.3 ± 0.1 ab	0.6 ± 0.3 ab	0.4 ± 0.1 ab	0.7 ± 0.2 a	0.04	14.35
**WDI (min)[a]**										
**Probe**	367.8 ± 19.6 a	304.9 ± 30.2 abc	339.1 ± 22.6 ab	298.5 ± 21.9 bc	225.2 ± 35.0 c	305.0 ± 27.5 ac	302.7 ± 30.6 ac	356.5 ± 22.9 ab	0.04	14.88
**np**	112.2 ± 19.6 a	175.1 ± 30.2 ab	140.9 ± 22.6 a	181.5 ± 17.1ab	254.8 ± 35.0 b	178.3 ± 26.2 ab	177.3 ± 30.6 ab	123.5 ± 22.9 a	0.03	15.15
**C**	155.4 ± 18.3 a	163.1 ± 17.0 a	142.7 ± 18.4 a	178.6 ± 17.0 a	140.5 ± 28.5 a	158.1 ± 20.7 a	154.3 ± 27.8 a	142.1 ± 18.1 a	0.72	0.64*
**G**	99.1 ± 28.5 ac	50.5 ± 13.0 abd	108.5 ± 19.9 c	60.5 ± 13.7 ac	17.2 ± 6.2 b	66.4 ± 21.1 ac	40.9 ± 15.5 ab	114.3 ± 26.7 dc	< 0.01	24.10
**F**	48.6 ± 18.3 a	23.6 ± 7.1 ac	48.4 ± 13.3 a	52.7 ± 14.3 a	2.0 ± 1.0 bc	21.2 ± 6.9 a	21.1 ± 12.6 ac	6.2 ± 2.7 b	< 0.01	29.56
**pd**	4.7 ± 0.7 ab	5.0 ± 1.2 abd	3.5 ± 0.7 acd	5.9 ± 0.7 b	2.5 ± 1.0 c	3.8 ± 0.9 ac	3.0 ± 0.9 dc	3.8 ± 0.8 abd	< 0.01	21.29
**E1**	9.5 ± 4.3 a	7.8 ± 3.8 a	7.9 ± 4.4 a	5.2 ± 4.9 a	3.8 ± 3.6 a	13.7 ± 5.9 a	19.7 ± 7.6 a	12.6 ± 6.4 a	0.11	11.81
**E2**	55.2 ± 19.2 a	59.9 ± 24.4 a	31.6 ± 15.6 a	4.3 ± 3.5 b	61.7 ± 27.8 a	42.4 ± 18.4 a	66.8 ± 25.0 a	81.4 ± 23.1 a	0.04	14.70
**Total duration of E**	64.7 ± 21.3 a	67.7 ± 25.0 a	39.5 ± 18.4 a	9.5 ± 6.3 b	65.6 ± 30.1 a	56.1 ± 21.6 a	86.5 ± 28.0 a	94.0 ± 23.5 a	0.04	19.92

[a] **NWEI,** number of waveform events per insect; **WDI,** total waveform duration (min) per insect; [b] Statistical comparisons between treatments for each parameter were made by Tukey test (*****) for Gaussian distribution variables or Kruskal-Wallis H-test for non-Gaussian distribution variables. Underline-type indicates significant differences ($P < 0.05$) (Backus et al., 2007).

Table S2. Mean (± SEM) of sequential EPG variables for 8-h recordings of the probing behavior of *Melanaphis sacchari* on sugarcane cultivars infected with sugarcane yellow leaf virus (ScYLV).

**EPG- Sequential variables**	**5094** n = 20	**2562** n = 17	**5503** n = 20	**7569** n = 18	**Bio266** n = 17	**3127** n = 17	**5000** n=18	**6163** n=18	***P*-value**[a]	**H value**
Time to 1st probe from start of EPG	11.1 ± 3.7 a	21.0 ± 8.2 a	34.3 ± 11.5 a	24.6 ± 10.5 a	10.9 ± 3.8 a	17.1 ± 6.9 a	20.8 ± 13.0 a	17.9 ± 8.9 a	0.54	5.95
Time from start of EPG to 1st E	322.4 ± 35.7 a	339.9 ± 38.6 a	379.3 ± 29.9 a	439.5 ± 25.6 a	369.2 ± 40.0 a	344.7 ± 33.5 a	360.1 ± 33.9 a	292.8 ± 38.6 a	0.08	12.79
Time from 1st probe to 1st E	311.2 ± 36.3 a	318.8 ± 36.3a	344.9 ± 31.6 a	415.0 ± 28.8 a	358.3 ± 40.8 a	327.6 ± 32.9 a	339.3 ± 34.9 a	274.9 ± 41.4 a	0.16	10.58
Time from start of EPG 1st sustained E_2 (>10min)	400.9 ± 26.1 a	378.7 ± 33.9 a	446.9 ± 16.6a	454.3 ± 22.3 a	410.8 ± 32.3 a	408.8 ± 28.9 a	391.7 ± 32.1 a	359.9 ± 30.4 a	0.10	12.00
Time from start of EPG to 1st E_2	350.8 ± 32.2 a	342.2 ± 37.8 a	394.4 ± 28.3 a	440.2 ± 25.6 a	387.0 ± 37.5 a	373.4 ± 32.8 a	364.5 ± 32.7 a	315.2 ± 34.2 a	0.12	11.38

[a]Statistical comparisons between treatments for each variable were made by non-parametric Kruskal-Wallis test. Underline-type indicates significant differences ($P < 0.05$).

Table S3. Proportion of *Melanaphis sacchari* that produced a specific waveform type (PPW) on sugarcane cultivars infected with sugarcane yellow leaf virus (ScYLV) during 8-h recording.

**Waveform**	**5094**	**2563**	**5503**	**7569**	**Bio266**	**3127**	**5000**	**6163**	***P*-value[b]**	**X^2**
**F**	16/20 a (80.0%)	11/17 a (64.7%)	12/20 a (60.0%)	13/18 a (72.2%)	4/17 b (23.5%)	13/17 a (76.5%)	4/18 b (22.2%)	5/18 b (27.8%)	< 0.01	31.011
**E1**	13/20 a (65.0%)	10/17 a (58.8%)	9/20 a (45.0%)	4/18 a (22.2%)	6/17 a (35.3%)	10/17 a (58.8%)	10/18 a (55.6%)	12/18 a (66.7%)	0.09	12.250
**E2**	11/20 a (55.0%)	10/17 a (58.8%)	9/20 a (45.0%)	3/18 a (16.7%)	6/17 a (35.3%)	8/17 a (47.1%)	10/18 a (55.6%)	12/18 a (66.7%)	0.09	12.372
**E2>10 min**	7/20 a (35.0%)	8/17 a (40.0%)	4/20 a (20.0%)	1/18 b (5.5%)	5/17 a (29.4%)	6/17 a (35.3%)	8/18 a (44.0%)	11/18 a (61.1%)	0.01	16.355

[a]EPG waveforms: (F) derailed stylet mechanics, (E1) salivation in phloem sieve elements, (E2) phloem sap ingestion. [b]Proportion followed by the same letter, in the same row, do not differ significantly (*P*-value>0.05) using chi-square (X^2) test for pairwise comparisons.
